# NOVA1 induction by inflammation and NOVA1 suppression by epigenetic regulation in head and neck squamous cell carcinoma

**DOI:** 10.1038/s41598-019-47755-8

**Published:** 2019-08-02

**Authors:** Eun Kyung Kim, Yoon Ah Cho, Mi-kyoung Seo, Hyunmi Ryu, Byoung Chul Cho, Yoon Woo Koh, Sun Och Yoon

**Affiliations:** 1 Department of Pathology, Severance Hospital, Yonsei University College of Medicine, Seoul, 03722 South Korea; 2 Department of Pathology, National Health Insurance Service Ilsan Hospital, Goyang, 10444 South Korea; 3 Department of Biomedical Systems Informatics, Brain Korea 21 PLUS Project for Medical Science, Yonsei University College of Medicine, Seoul, 03722 South Korea; 4 Division of Medical Oncology, Department of Internal Medicine, Yonsei Cancer Center, Severance Hospital, Yonsei University College of Medicine, Seoul, 03722 South Korea; 5 Department of Otorhinolaryngology, Severance Hospital, Yonsei University College of Medicine, Seoul, 03722 South Korea

**Keywords:** Cancer microenvironment, Cancer microenvironment, Head and neck cancer

## Abstract

Neuro-oncological ventral antigen 1 (NOVA1) is known as a neuron-specific pre-mRNA binding splicing factor. Previously, it was shown to be highly upregulated in T lymphocytes, as well as fibroblasts/stromal spindle cells, in tertiary lymphoid tissues formed by the benign immune-inflammatory process, while it was frequently downregulated in tumor cells and other cells within the tumor microenvironment. Here, we sought to identify the mechanisms of NOVA1 modulation in head and neck squamous cell carcinoma (HNSCC). NOVA1 was induced by inflammatory-immune signals within the tumor microenvironment and was suppressed by epigenetic dysregulation, such as that with miR-146. We found attenuated expression of NOVA1 to be associated with non-oropharynx sites such as oral cavity, hypopharynx, and larynx, human papilloma virus (HPV)-negative SCC defined by immunohistochemistry for p16^INK4a^ expression, fewer tumor infiltrating lymphocytes, and poor patient outcomes. Moreover, changes were discovered in epithelial mesenchymal transition-associated markers according to NOVA1 status. This study provides some insights to the underlying mechanism of NOVA1 regulation and suggests that NOVA1 may serve as a prognostic biomarker and potential therapeutic target for HNSCC in the future.

## Introduction

Neuro-oncological ventral antigen 1 (NOVA1) is a neuron-specific pre-mRNA binding splicing factor and is necessary for the development of the motor system and the survival of motor neurons. Recent studies have shown that NOVA1 is enriched in normal fibroblasts and activated T cells^1–5^ and plays a role in various cancers^6–8^. Through our research, we have discovered that NOVA1 is strongly expressed in most fibroblasts/stromal spindle cells and in only a small number of T lymphocytes within normal physiologic secondary lymphoid tissue; meanwhile, it appears to be highly upregulated in T lymphocytes, as well as fibroblasts/stromal spindle cells, in tertiary lymphoid tissues formed by benign immune-inflammatory processes, such as *Helicobacter pylori*-associated gastritis. Within the tumor microenvironment of gastric cancers, we have observed progressive loss of NOVA1 expression in T lymphocytes, stromal spindle cells, and tumor cells with more advanced stages of gastric cancer and have found decreased NOVA1 expression in these cells to be related to poor patient prognosis. Also, we have reported that miR-146b-5p may target NOVA1 and may lead to NOVA1 suppression^9–11^. Meanwhile, through preliminary experiments, we have discovered that NOVA1 is highly expressed in both tumor cells and microenvironment cells in human papilloma virus (HPV)-related oropharyngeal squamous cell carcinoma (SCC), but decreased in HPV-negative head and neck squamous cell carcinoma (HNSCC) (Supplementary Fig. S1). These findings suggest that inflammatory and immune reactions in response to HPV infection might be involved in the induction of NOVA1 within the tumor microenvironment.

According to comprehensive genetic analyses using The Cancer Genome Atlas Network (TCGA, https://cancergenome.nih.gov) and the Catalogue Of Somatic Mutations in Cancer (COSMIC, http://cancer.sanger.ac.uk/cosmic), genetic mutations and epigenetic hypermethylation eliciting NOVA1 dysregulation appear to be rare in most cancers. Despite marked variations in expression levels of NOVA1 in HNSCC, recent comprehensive studies have revealed that genetic alteration of *NOVA1* is very rare, at a frequency of about 2% (Supplementary Fig. S2). From this and our previous study^11^, we conjectured that epigenetic regulation, specifically with microRNAs (miRNA), may be involved in the dysregulation of NOVA1 in HNSCC.

In the present study, we sought to determine whether NOVA1 is induced by inflammatory signals and epigenetically suppressed within the tumor microenvironment in HNSCC.

## Results

### NOVA1 expression in tumor cells upon HPV E6/E7 transfection

**Figure 1:**
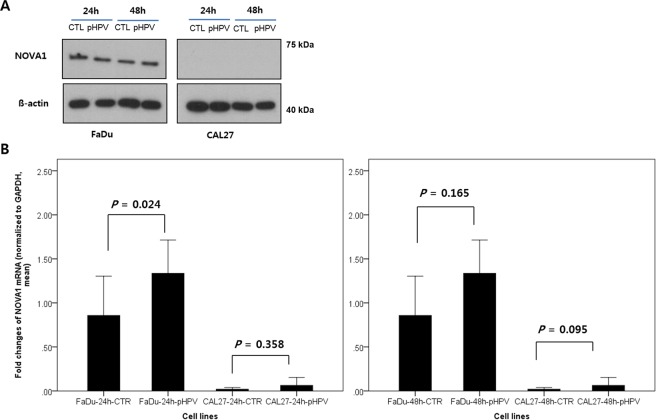
NOVA1 expression after transfection of plasmid p1321 HPV-16 *E6/E7*. (**A**) In Western blot analysis, NOVA1 protein expression was detected in FaDu cells, but not in CAL27 cells. Transfection of HPV type 16 *E6/E7* genes into FaDu and CAL27 did not induce a significant change in NOVA1 protein expression. (**B**) Generally, no significant changes in NOVA1 mRNA expression were observed upon transfection of HPV-16 *E6/E7* genes into FaDu and CAL27 cells; a slight increase in mRNA was noted after 24 hours of transfection into FaDu cells. Fold changes in NOVA1 mRNA values were calculated based on NOVA1 levels of FaDu-CTR at 24 h and 48 h. Compared to NOVA1 mRNA levels in FaDu cells, those in CAL27 cells were very low. (CTL, controls with transfected empty vector; pHPV, cells with transfected plasmid HPV-16 *E6/E7*).

Western blot analysis revealed NOVA1 expression in FaDu cells, but not in CAL27 cells. While p1321 HPV-16 *E6/E7* plasmid was successfully transfected into FaDu and CAL27 cells (Supplementary Fig. S3), it did not induce a significant change in NOVA1 protein levels (Fig. 1A and Supplementary Fig. S4). Real-time PCR analysis of NOVA1 mRNA expression generally showed no significant changes therein upon transfection of HPV-16 *E6/E7* genes into FaDu and CAL27 cells, although there was a slight increase in NOVA1 mRNA after 24 hours of transfection into FaDu cells (Fig. 1B).

### NOVA1 induced by poly(dA:dT) and suppressed by miR-146

**Figure 2:**
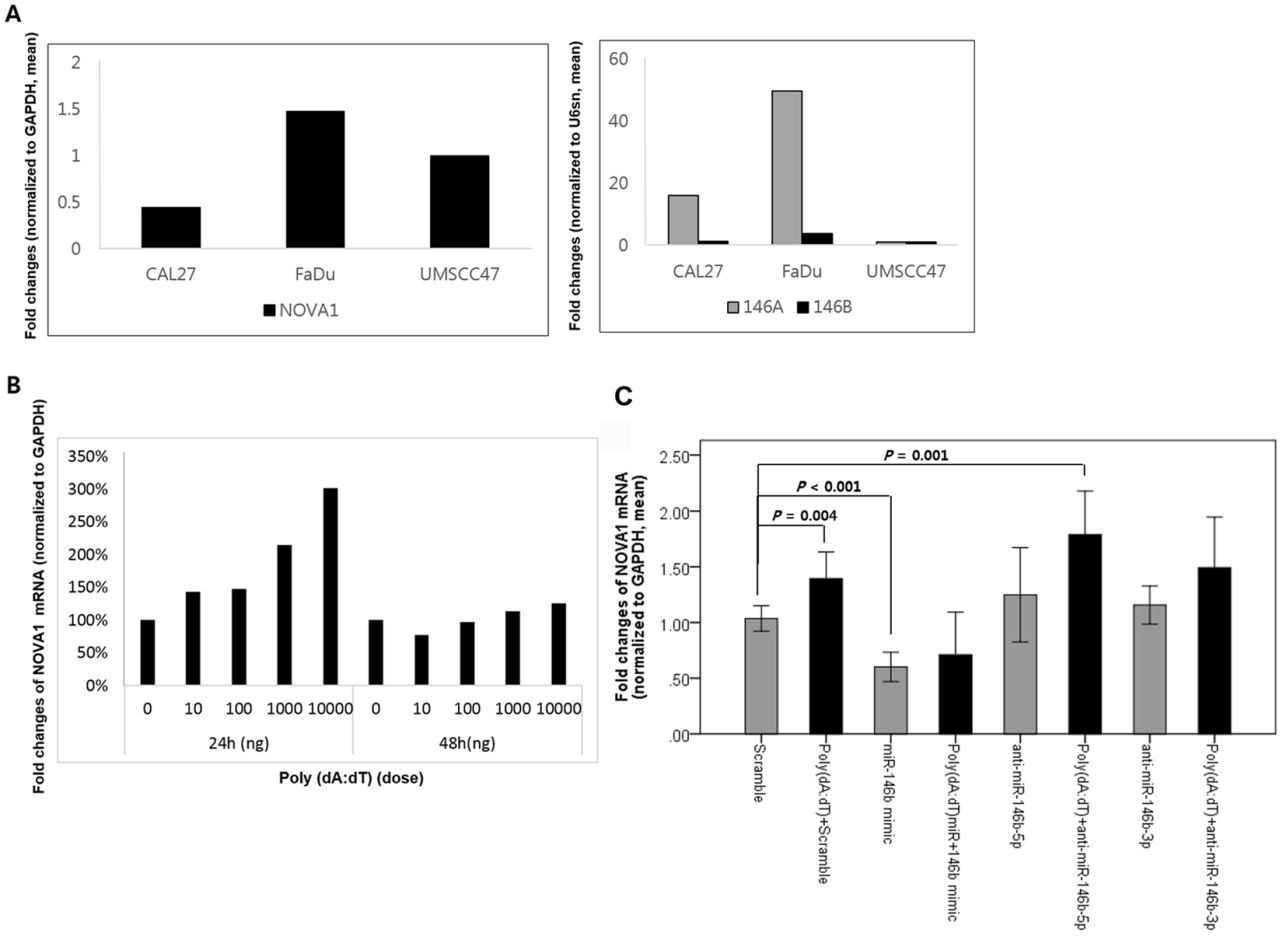
Endogenous and post-transfection expression levels of NOVA1. (**A**) Endogenous expression of NOVA1 protein and predicted target miRs in the HPV-negative non-oropharynx SCC cell lines FaDu and CAL27 and the HPV-positive oropharynx SCC cell line UMSCC47. FaDu cells show the highest endogenous expression levels of NOVA1, miR-146a-5p, and miR-146b-5p. Fold changes in each molecule were calculated in comparison to UMSCC47. (**B**) After 24 hours of transfection of poly(dA:dT) double-stranded DNA in FaDu cells, a dose-dependent increase in NOVA1 expression was noted. (**C**) After 24-hour transfection into FaDu cells, NOVA1 levels were the lowest when treating with miR-146b mimic, but the highest when treating with poly(dA:dT) + anti-miR-146b-5p. When treating with miR-146b mimic, adding poly(dA:dT) did not significantly increase NOVA1 expression. Fold changes in NOVA1 were calculated in comparison to cells treated with scramble.

Endogenous expression levels of predicted target miRs, including hsa-miR-146a-5p, -146b-5p, -181a, -181b, -181c, -181d, -27a, and -27b, were compared among the HPV-negative non-oropharynx SCC cell lines FaDu and CAL27 and the HPV-positive oropharynx SCC cell line UMSCC47 (Supplementary Fig. S5). FaDu cells, which endogenously express NOVA1, exhibited greater expression of these miRs, especially miR-146a-5p and miR-146b-5p, compared to CAL27 cells, which showed very low NOVA1 expression (Fig. 2A).

After 24 hour-transfection of poly(dA:dT) double-stranded DNA into FaDu cells, a dose-dependent increase in NOVA1 expression was noted (Fig. 2B). Confirming the suppressive effect of miR-146b on NOVA1, FaDu cells showed decreased NOVA1 expression after 24 hours of transfection with miR-146b mimic (Fig. 2C; scramble versus miR-146b mimic; p < 0.001). Transfecting inhibitors of miR-146b (anti-miR-146b-5p or anti-miR-146b-3p) only slightly increased NOVA1 levels in FaDu cells; the difference was not statistically significant (scramble versus anti-miR-146b-5p, p = 0.106; scramble versus anti-miR-146b-3p, p = 0.067; Fig. 2C). Meanwhile, both treatment with poly(dA:dT) alone and treatment with poly(dA:dT) and anti-miR-146b-5p or poly(dA:dT) and anti-miR-146b-3p significantly increased NOVA1 expression in FaDu cells (Fig. 2C; scramble versus poly(dA:dT) + scramble; p = 0.004; scramble versus poly(dA:dT) + anti-miR-146b-5p, p = 0.001; scramble versus poly(dA:dT) + anti-miR-146b-3p, P = 0.014; anti-miR-146b-5p versus poly(dA:dT) + anti-miR-146b-5p, p = 0.015; anti-miR-146b-3p versus poly(dA:dT) + anti-miR-146b-3p, p = 0.041). Treatment with miR-146b mimic alone however, did not significantly increase NOVA1 levels above treatment with miR-146b mimic and poly(dA:dT) in FaDu cells (Fig. 2C; miR-146b mimic versus poly(dA:dT) + miR-146b mimic, p = 0.305). Overall, NOVA1 levels were the lowest when treating FaDu cells with miR-146b mimic and highest when treating FaDu cells with poly(dA:dT) + anti-miR-146b-5p.

### Expression of miR-146 and NOVA1 in tumor and microenvironment cells in HNSCC tissue samples

**Figure 3:**
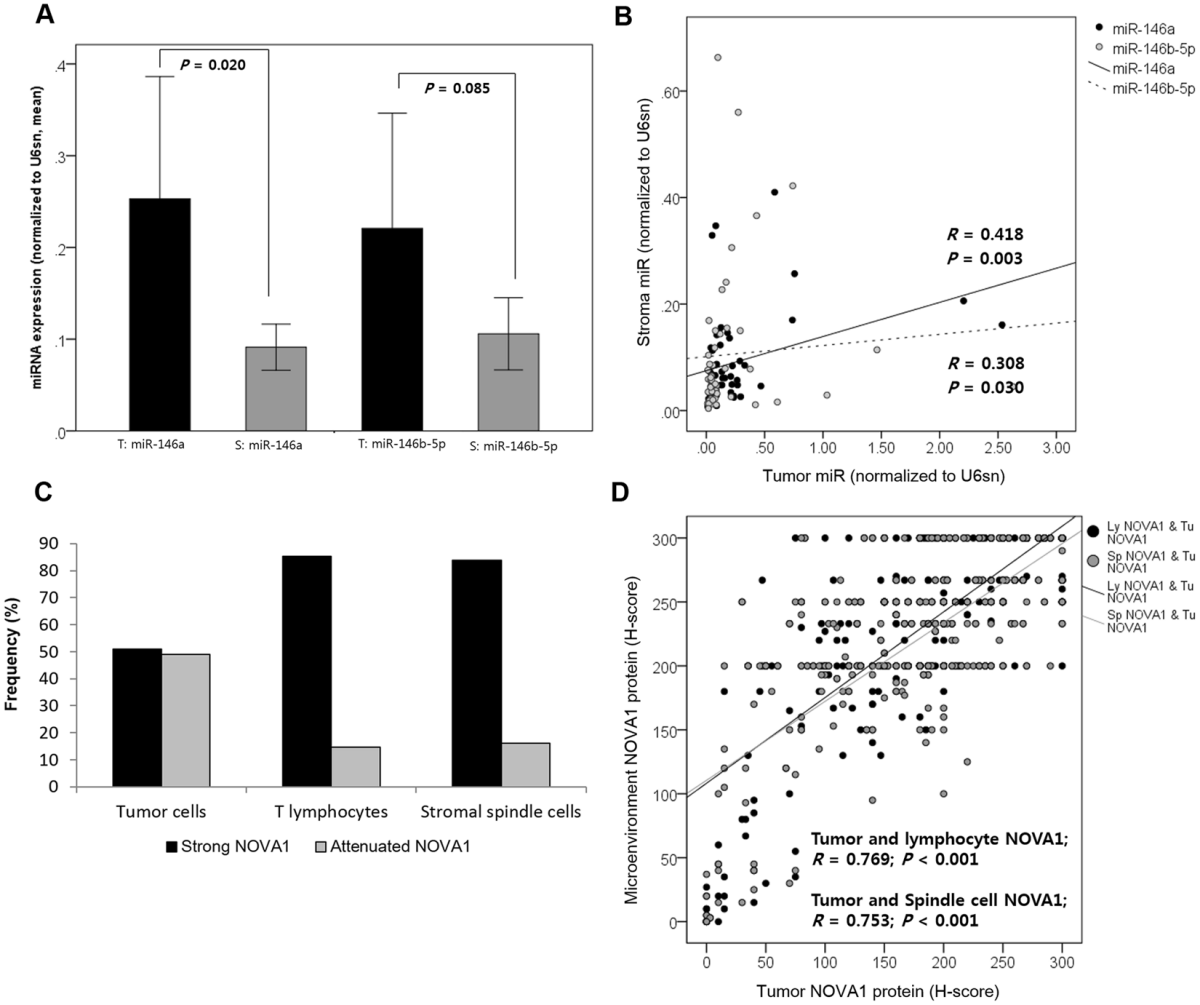
Expression of miR-146 and NOVA1 in tumor cells and microenvironment cells from HNSCC tissue samples. (**A**) Expression of miR-146 in tumor areas (T) and stromal areas (S) of HNSCC tissue samples (n = 50). General expression levels of miRs, especially miR-146a, were higher in tumor areas than in stromal areas. (**B**) Levels of miR-146 in tumor and stroma areas were positively correlated. (**C**) Attenuated NOVA1 expression, reflected as an H-score < 200, indicating loss of NOVA1 in more than one-third of cells, were more frequently observed in tumor cells than in lymphocytes and stromal spindle cells. (**D**) Generally, tumor NOVA1 expression status was correlated with that of T lymphocytes and stromal spindle cells (P < 0.001, respectively).

In HNSCC samples from 50 randomly selected patients, hsa-miR-146a-5p and -146b-5p were investigated in tumor and adjacent stromal areas. General expression levels of miRs, especially miR-146a, were higher in tumor areas than in stromal areas (Fig. 3A). MiR levels in tumor and stroma tissue were positively correlated, although the correlation strength was fair (has-miR-146a-5p, r = 0.418, p = 0.003; has-miR-146b-5p, r = 0.308, p = 0.003; Fig. 3B).

NOVA1 expression varied in tumor cells and lymphocytes and stromal spindle cells from the tissue microenvironment of 396 HNSCC specimens. Attenuated NOVA1 expression, which showed an H-score < 200 and indicated a loss of NOVA1 in more than one-third of the cell population, was more frequent in tumor cells than in lymphocytes and stromal spindle cells (Fig. 3C and Supplementary Fig. S6). Generally, NOVA1 expression status in tumor cells was correlated with that of T lymphocytes and stromal spindle cells (tumor cells and T lymphocytes, r = 0.769, p < 0.001; tumor cells and stromal spindle cells, r = 0.753, p < 0.001; Fig. 3D).

### NOVA1 expression in association with anatomical sites, HPV infection status, T cell infiltration, epithelial mesenchymal transition markers, and patient outcomes

**Figure 4:**
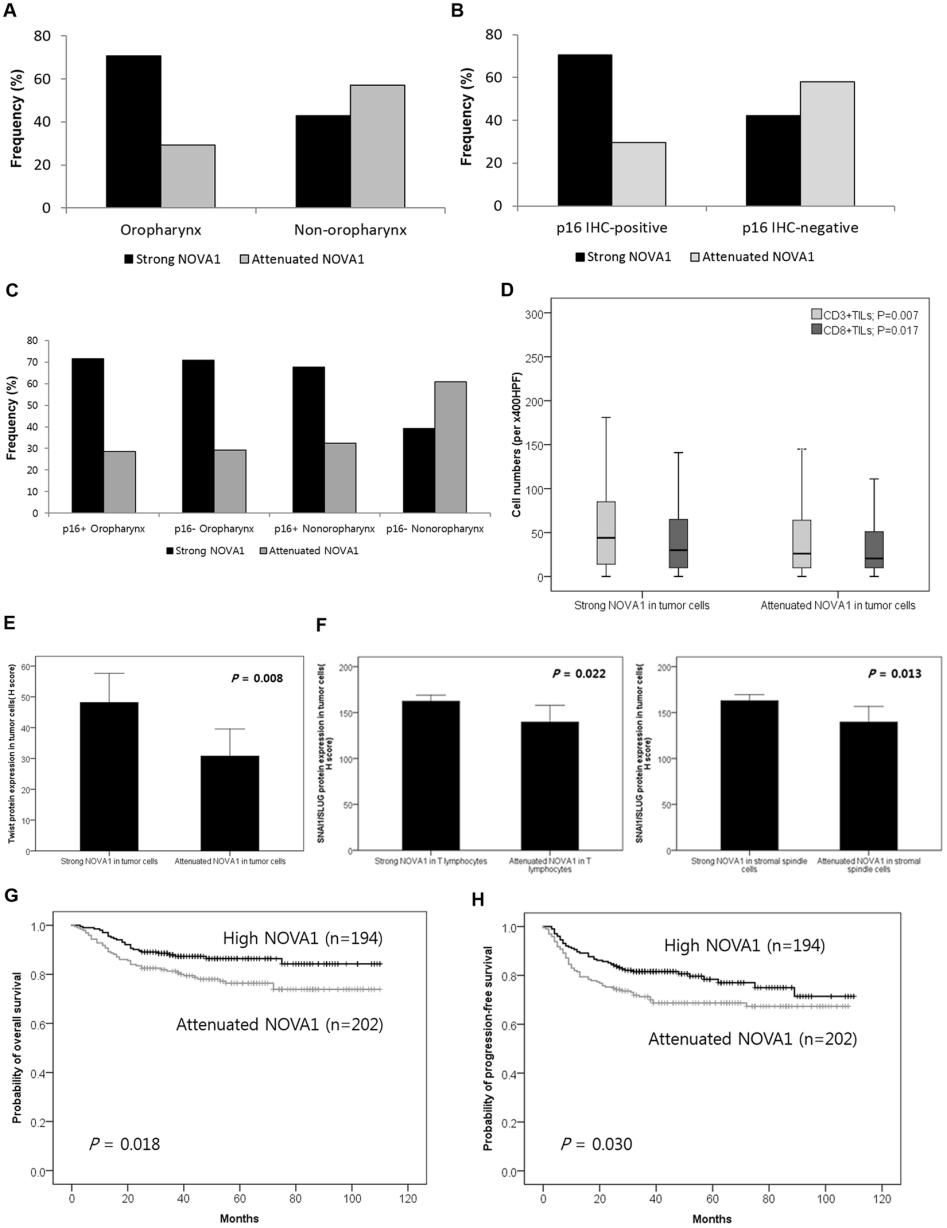
NOVA1 expression in association with anatomical sites, HPV infection status, T cell infiltration, EMT markers, and patient outcomes. (**A**,**B**) Attenuated NOVA1 expression (H-score < 200) in tumor cells was more frequent in non-oropharynx SCC and p16 (HPV)-negative SCC (P < 0.001, respectively). (**C**) Attenuated expression of NOVA1 in tumor cells was most frequently observed in p16-negative non-oropharynx SCC (P < 0.001). (**D**) Specimens showing attenuated NOVA1 among tumor cells showed lower levels of CD3+ or CD8+ TILs than those with strong NOVA1 expression. Average cell numbers of T lymphocytes were counted at x400 magnification (x400 HPF). (**E**) Twist expression was higher in tumor cells from specimens with high NOVA1 expression. (**F**) SNAI1/SLUG expression in tumor cells showed significant differences according to NOVA1 expression status in T lymphocytes and stromal spindle cells. (**G**,**H**) Attenuated NOVA1 expression in tumor cells was associated with inferior overall survival and progression-free survival rates.

Attenuated expression (H-score < 200) of NOVA1 in tumor cells was frequent in non-oropharynx SCC such as SCC arising in oral cavity, hypopharynx, larynx, and p16 (HPV)-negative SCC (p < 0.001, respectively; Fig. 4A,B), and it was most frequently observed in p16-negative non-oropharynx SCC, when comparing oropharynx SCC and/or HPV-positive cases (p < 0.001; Fig. 4C). Regarding TILs, which are observed within tumor cell nests, specimens showing attenuated NOVA1 expression in tumor cells showed lower cell densities of CD3+ or CD8+ TILs than those showing strong NOVA1 expression (Fig. 4D). A marker of epithelial mesenchymal transition (EMT), Twist expression in tumor cells was related to NOVA1 expression status in tumor cells, but not to NOVA1 status in stromal spindle cells/fibroblasts or T lymphocytes (Fig. 4E; Supplementary Fig. S7). SNAI1/SLUG expression (another marker of EMT) in tumor cells was related to NOVA1 expression status in T lymphocytes and stromal spindle cells/fibroblasts, but not to NOVA1 status in tumor cells (Fig. 4F; Supplementary Fig. S7).

Regarding associations with patient outcomes, attenuated NOVA1 expression in tumor cells was associated with inferior OS (Fig. 4G, p = 0.018) and PFS in Kaplan-Meier analysis (Fig. 4H, p = 0.030). In multivariate Cox regression analysis, attenuated NOVA1 expression in tumor cells was identified as an independent poor prognostic factor for OS (Hazard ratio = 2.104, p = 0.005) and PFS (Hazard ratio = 1.599, p = 0.03), as were other clinicopathologic factors, such as older age, non-oropharynx site, advanced pathologic T stage (pT stage 3–4), advanced pathologic N stage (pN stage 2–3), and lower cell density of tumor infiltrating CD8 + cytotoxic T cells (Supplementary Tables S5 and S6).

### Microenvironment Cell Populations-counter analysis

**Figure 5:**
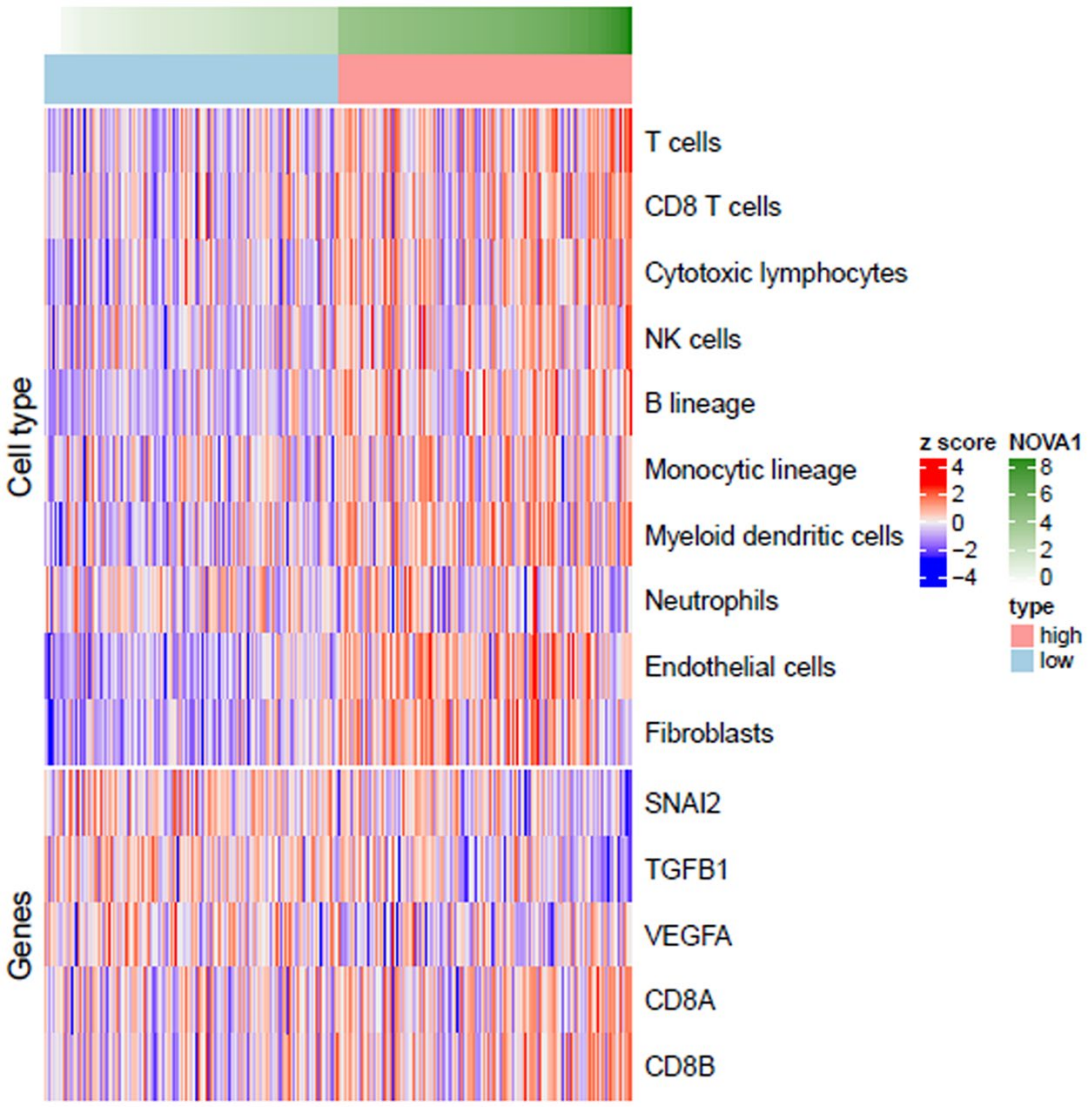
Microenvironment Cell Populations-counter analysis. Z-score transformed values of log2 (normalized rsem + 1) values of genes and MCP-counter values were used to identify differences between groups for cell type abundance, inflammation-related genes, and EMT-related genes. Lower quantities of immune cells and stromal cells, downregulation of CD8+ T cell-related genes, and upregulation of SNAI2 and TGFB1 were related to low NOVA1 expression.

Microenvironment Cell Populations-counter (MCP-counter) analysis using a gene set for HNSCC generated from TCGA (https://cancergenome.nih.gov, n = 348) indicated that high NOVA1 expression is significantly correlated with greater abundance of immune cells, including T cells, CD8+ T cells, cytotoxic lymphocytes, NK cells, B cells, monocytes, and myeloid dendritic cells, as well as stromal cells of fibroblasts and endothelial cells. Upregulation of the CD8+ T cell-related genes *CD8A* and *CD8B* was significantly related to high NOVA1 expression (Fig. 5; Supplementary Table S7), as were upregulation of *TWIST* and *SNAI1* and downregulation of *SNAI2 (SLUG)* and *TGFB1* (Fig. 5; Supplementary Table S8). In summary, lower abundances of immune and stromal cells, downregulation of CD8+ T cell-related genes, downregulation of *TWIST* and *SNAI1*, and upregulation of *SNAI2* and *TGFB1* were all found to be related to low NOVA1 expression (Fig. 5; Supplementary Tables S7 and S8; Supplementary Fig. S8).

## Discussion

In the present study, we first sought to determine whether NOVA1 is induced in tumor cells by inflammatory signals within the microenvironment of HNSCC. Oropharynx SCC arises within lymphoid and immune cell-rich microenvironments (palatine tonsils and base of the tongue) and is primarily associated with HPV infection. Non-oropharynx SCC such as SCC of oral cavity, hypopharynx, and larynx, however, arises from an immune cell-poor tissue microenvironment and is generally unrelated to HPV infection. Although the lymphoid and immune cell structures of the oropharynx are physiologically formed as secondary lymphoid structures, inflammatory stimuli in response to HPV infection are thought to induce NOVA1 expression in tumor cells and the surrounding microenvironment. Nonetheless, in non-oropharynx SCC, NOVA1 could still be induced in tumor cells and the surrounding microenvironment upon formation of tertiary lymphoid structures within the tumor microenvironment as an immune response to tumor growth^12–16^. As expected, in the present study, most oropharynx SCCs showed strong NOVA1 expression in tumor cells, while non-oropharynx SCC frequently showed attenuated expression. Likewise, HPV (p16 immunohistochemistry)-positive SCC mostly exhibited strong NOVA1 expression, while HPV-negative SCC frequently showed attenuated expression in tumor cells. Such high NOVA1 expression in oropharynx SCC may be related to an abundance of lymphoid structures within the tissue microenvironment.

As stated above, most oropharynx SCCs show HPV positivity. Thus, immune reactions within the lymphoid structures, which are activated by HPV infection, might stimulate NOVA1 induction. HPV type 16 is the most prevalently detected virus type in HPV-mediated oropharynx SCC, accounting for about 80% of all HPV-positive SCC cases, and HPV *E6* and *E7* are the most potent oncogenes affecting the tumorigenesis of HPV-mediated tumors^17^. In the present study, transfection of HPV 16 *E6/E7* genes to originally HPV-negative SCC cells did not significantly induce NOVA1 in tumor cells. Accordingly, we deemed that HPV infection, triggering immune reactions within the surrounding microenvironment, is only indirectly involved in NOVA1 induction.

As a limitation of our study, a single tumor cell line could not fully represent actual tissue conditions, comprising activated inflammatory and lymphoid structures formed within the tissue microenvironment. To address this limitation, we sought to simulate immune signals by treating cells with poly(dA: dT). Transfected poly(dA: dT) is recognized by several cytosolic DNA sensors, activates immune responses, and triggers the formation of the inflammasome^18–21^. Indeed, by mimicking inflammatory signals, poly(dA:dT) upregulated NOVA expression in the present study. In line with this result, we noted higher infiltrating cell densities of CD3+ T lymphocytes and CD8+ cytotoxic T lymphocytes in HNSCC specimens showing strong NOVA1 expression among tumor cells, compared to specimens showing attenuated NOVA1 expression. MCP-counter analysis using gene sets for HNSCC from TCGA also indicated that high NOVA1 expression may be related to increases in immune cells, such as CD8+ T cells, and stromal cells, such as fibroblasts. Overall, these findings support our hypothesis that inflammatory and immune signals within the microenvironment may induce NOVA1 upregulation.

Despite our meaningful results, the biological significance of NOVA1 upregulation in cancer remains uncertain. To identify possible roles for NOVA beyond its known role as a neuron-specific pre-mRNA binding splicing factor, we focused on EMT, since NOVA1 upregulation seems to occur by interacting with microenvironment inflammatory signals and since NOVA1 is known to be enriched in normal fibroblasts^5^. In HNSCC tissue samples, we found Twist and SNAI1 expression (EMT markers) to be related to NOVA1 status in tumor cells, as well as stromal spindle cells and T lymphocytes. Moreover, MCP-counter analysis using the gene set of HNSCC from TCGA also indicated that NOVA1 expression is related to upregulation or downregulation of EMT signature genes, including *SNAI1, TWIST, SNAI2*, and *TGFB1*. Although further studies of direct signal pathways are needed, these findings suggest that changes in NOVA1 expression in tumor cells or the tissue microenvironment may affect EMT and that NOVA1 may act as a signaling factor between tumor cells and interacting stromal cells.

Regarding the clinical implications of NOVA1 in HNSCC, we discovered that attenuated NOVA1 expression in tumor cells is related to poor patient prognosis via univariate and multivariate survival analysis. This suggests that NOVA1 suppression might be related to an aggressive cancer cell phenotype. As a mechanism of NOVA1 suppression, we suspected that epigenetic regulations could affect genetically normal lymphocytes and stromal spindle cells. Several studies have found that altered miRNA expression likely occurs in the tumor microenvironment as the result of crosstalk among stromal spindle cells and immune cells through various signaling autocrine and/or paracrine methods^22^. In HNSCC tissue samples, we noted that variable expression of hsa-miR-146a-5p and miR-146b-5p, which are predicted to target NOVA1, was positively correlated between tumor and stromal areas: the expression levels of individual miRs were relatively higher in tumor areas than in stromal areas, suggesting that a major source of upregulated miRs may be tumor cells. To confirm NOVA1 inhibition by miRs *in vitro*, we experimented on FaDu cells, because this cell line shows higher expression of miR-146 and NOVA1. Because the sequences of miR-146a-5p and miR-146b-5p were very similar and their predicted target sites of NOVA1 were identical (-AGUUCUC-: Supplementary Fig. S9), transfection experiments were performed with miR-146b mimic and anti-miR-146b inhibitors. In doing so, we found that miR-146 (miR-146a and miR-146b) could suppress NOVA1 in tumor cells, even in the presence of inflammatory signals simulated by poly(dA:dT) transfection. When simultaneously treating cells with poly(dA:dT) and blocking miR-146 with anti-miR-146b-5p and anti-miR-146b-3p, NOVA1 expression was increased. From these findings, we discerned that miR-146 may be potent suppressors of NOVA1, even in the presence of inflammatory signals. These results could reflect a mechanism by which NOVA1 is epigenetically attenuated in tumor cells, even when immune-inflammatory lymphoid structures are present, within the tumor microenvironment.

In the process of cancer development and progression, HNSCCs adapt several mechanisms of immune escape, such as T cell tolerance, inhibition of inflammatory cytokines, and exhaustion of effective cytotoxic T cell function within the tumor microenvironment^23–25^. In this study, we found that NOVA1 is induced by inflammatory-immune signals within the tissue microenvironment and is suppressed by epigenetic dysregulation, potentially by miR-146. Although epigenetic suppression of NOVA1 and impaired immune surveillance within the tissue microenvironment according to tumor progression should be investigated further, the present study provides some insights into the underlying mechanism of NOVA1 regulation and raises the translational potential of NOVA1 as a prognostic biomarker and therapeutic target for HNSCC in the future.

## Methods

### Cell lines and culture conditions

In the present study, the HPV-negative HNSCC cell lines FaDu (ATCC^®^ HTB-43^™^, pharynx SCC) and CAL27 (ATCC^®^ CRL-2095^™^, tongue SCC) were used. As a HPV-positive oropharynx SCC cell line, UMSCC47 (ATCC^®^ CRL-3239™, base of tongue SCC) was used. FaDu cells were maintained in RPMI-1640 medium (22400-089, Gibco; Life Technologies, CA, USA) supplemented with 10% FBS (6000-044, Gibco; Life Technologies). CAL27 and UMSCC47 cells were maintained in DMEM medium (SH30243.01, HyClone; Thermo Scientific, Logan, UT, USA) supplemented with 10% FBS (6000-044, Gibco; Life Technologies).

### Clinical samples

From the database of Severance Hospital Cancer Registry Data, Seoul, South Korea, cases of HNSCC were retrieved. Affected anatomical sites included the oropharynx (tonsils, base of tongue, soft palate, and oropharynx) and non-oropharynx. Non-oropharyngeal sites included oral cavity (anterior two-thirds of the tongue, mouth floor, hard palate, buccal mucosa, and unspecified oral cavity), hypopharynx, larynx (lingual surface of the epiglottis, glottis, supraglottis, subglottis, and larynx), and nasal cavity/paranasal sinuses. Carcinomas of nasopharynx, ear cavity, salivary gland, or other anatomical sites were excluded from the present study. Formalin-fixed paraffin-embedded tissue (FFPE) specimens from 116 consecutive oropharyngeal SCC and 280 consecutive non-oropharyngeal SCC patients were included: these patients underwent surgical resection with a curative aim from 2005 to 2012 at Severance Hospital Seoul, South Korea. Specimens that underwent decalcification were excluded for accurate immunohistochemical analysis. Part of the present cohort had been included in our previous studies^26–30^. Information on various clinical factors, including age at operation, sex, smoking, and alcohol consumption, was obtained by reviewing medical records. Pathologic factors, including lymphovascular invasion, perineural invasion, pathologic TNM staging according to the 7^th^ American Joint Committee on Cancer, and tumor classification by the World Health Organization system^31,32^, were obtained from slide review by three individual pathologists (E.K. Kim, Y.A. Cho, and S.O. Yoon). The median follow-up period was 50 months (range from 1 to 110 months). Clinicopathologic characteristics are described in Supplementary Table S1. This study was approved by the Institutional Review Board of Severance Hospital (Protocol No. 4-2015-0954). A written informed consent of the patients was obtained, samples and clinical information were anonymized. All experiments were performed in accordance with relevant guidelines and regulations (including all laboratory and biosafety regulations).

### Transfection of HPV E6/E7 gene and poly(dA:dT)

For transfection of the HPV E6/E7 gene and poly(dA:dT), six-well plate polycarbonate membrane transwell inserts (Corning Costar, Cambridge, MA, USA) were used. With the TransIT-X2® Dynamic Delivery System (MIR 6000; Cambridge Bioscience, Cambridge, UK), we transfected p1321 HPV-16 *E6/E7* plasmid (Addgene 292613, Cambridge, MA, USA) into each cell line, and after 24 and 48 hours, RNA was isolated.

Also, using the TransIT-X2® Dynamic Delivery System (MIR 6000; Cambridge Bioscience, UK), we transfected repetitive synthetic double-stranded DNA sequences of poly(dA-dT)•poly(dT-dA) (Sigma-Aldrich, MO, USA) into each cell line at a dose of 0, 10, 100, 1000, or 10000 ng. RNA was isolated 24 and 48 hours thereafter. All experiments were performed independently three or more times.

### Selection of candidate miRNAs targeting NOVA1 and transfection of miRNAs

Predicted target miRNA to NOVA1 were searched in a web-based database (http://www.microrna.org/microrna). We selected the first eight miRNAs with good support vector regression (SVR) values: miR-146a-5p, miR-146b-5p, miR-181a, miR-181b, miR-181c, miR-181d, miR-27a, and miR-27b.

Scrambled control miR (AccuTarget™ miRNA mimic Negative control; Bioneer, Daejeon, South Korea), hsa-miR-146b mimic (Bioneer), hsa-miR-146b-5p inhibitor (Bioneer), and hsa-miR-146b-3p inhibitor (Bioneer) were obtained and transfected at a concentration of 30 nM using the TransIT-X2® Dynamic Delivery System (MIR 6000; Cambridge). Co-transfection was performed with 100 ng of poly(dA:dT) and miR oligonucleotides of scramble miR, hsa-miR-146b mimic, hsa-miR-146b-5p inhibitor, or hsa-miR-146b-3p inhibitor. RNA was isolated after 24 hours of co-transfection. Experiments were performed independently three or more times.

### RNA isolation and quantitative real-time PCR (qRT-PCR)

RNA was isolated using RNeasy Plus Mini Kits (74134, Qiagen, Carlsbad, CA, USA), and cDNA was synthesized using cDNA synthesis kits (PB30.11 PCR Biosystems Ltd, Plymouth, PA, USA). Primers for HPV-16 *E6*, HPV-16 *E7*, *TWIST, SNAI1, NOVA1*, and *GAPDH* were manufactured (Integrated DNA Technologies, Coralville, IA, USA). The primer sequences are displayed in Supplementary Table S2. Using 2X qPCRBIO SyGreen Mix Lo-Rox (PB20.11 PCR Biosystems Ltd., Plymouth, PA, USA) as PCR reagent and the ABI 7500 Real-Time PCR System (Thermo Scientific, Logan, UT, USA), PCR was performed at 95 °C for 2 min; at 95 °C for 5 s and 60 °C for 30 s over 40 cycles; 95 °C for 15 s; 60 °C for 1 min; and 95 °C for 15 s. Experiments were performed independently three or more times.

### Quantification of miRNA in clinical samples of HNSCC

The expression patterns of candidate miRNAs in the microenvironment were analyzed in 50 FFPE specimens of HNSCC that were randomly selected and showed variable NOVA1 protein expression in tumor cells and stromal cells. Information on NOVA1 protein expression status in these 50 specimens is summarized in Supplementary Table S3. Tumor areas and surrounding stromal areas were separately dissected under a microscope. RNA, including microRNA, was isolated using miRNeasy FFPE Kits (Qiagen, Hilden, Germany). cDNA was synthesized using looped reverse transcription primers specific to individual miRNA species. Looped reverse transcription and forward and reverse primers for each microRNA species (hsa-miR-146a-5p, -146b-5p, -181a, -181b, -181c, -181d, -27a, and -27b, as well as a housekeeping gene, U6sn) are described in Supplementary Table S4. The expression of miRNA species was assayed quantitatively using a StepOnePlus Real-Time PCR instrument (Applied Biosystems, CA, USA) under the conditions of 94 °C for 2 min; 30 cycles of 94 °C for 15 s and 66 °C for 40 s; 94 °C for 15 s; 60 °C for 1 min; and 94 °C for 15 s. All samples were analyzed twice to confirm reproducibility.

### Immunohistochemistry and interpretation

Tissue microarray analysis was conducted by selecting two or three different tumor areas from 396 HNSCC samples. Core specimens 3 mm in diameter were obtained from donor tissue blocks and arranged in recipient TMA blocks using a trephine apparatus. Immunohistochemistry (IHC) was performed on 4-μm tissue microarray sections using a Ventana Bench Mark XT Autostainer (Ventana Medical Systems, Tucson, AZ, USA) and a LEICA BOND-III Autostainer (Leica Biosystems, Newcastle Upon Tyne, UK) according to the manufacturers’ protocols. The tested primary antibodies were as follows: p16 (RTU; Ventana), NOVA1 (1:500 dilution; Abcam, Cambridge, UK), CD3 (1:200 dilution; LabVision, Fremont, CA, USA), CD8 (RTU; clone C8/144B; Dako, Glostrup, Denmark), Twist (1:200 dilution; Abcam), and SNAI1/SLUG (1:200 dilution; Abcam).

**Figure 6:**
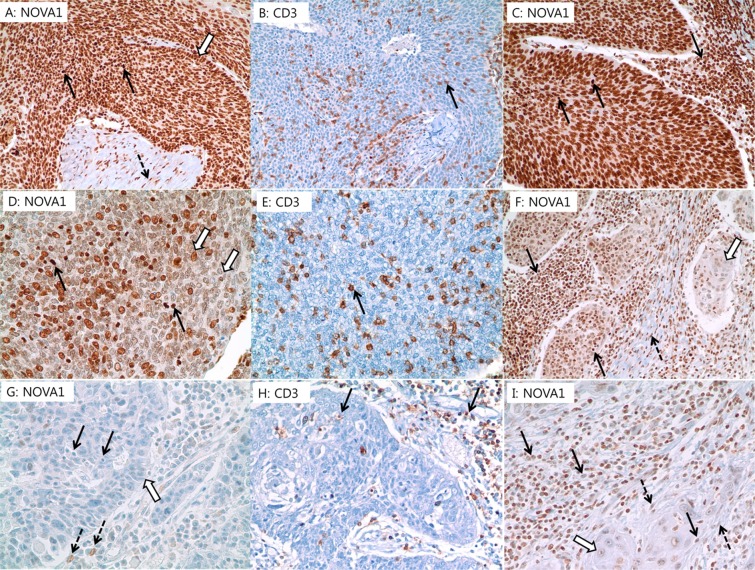
Representative cases of NOVA1 expression in tumor cells (empty arrow), T lymphocytes (black arrow), and stromal spindle cells (dotted arrow). (**A**,**B**) Almost all tumor cells, T lymphocytes within tumor cell nests, and stromal spindle cells exhibit strong NOVA1 expression. (**C**) Another case showing strong NOVA1 expression in tumor cells and T lymphocytes within tumor cell nests and peritumoral stroma. (**D**,**E**) Tumor cells show attenuated NOVA1 expression, while infiltrating T lymphocytes show strong NOVA1 expression. (**F**) Attenuated NOVA1 expression in tumor cells and strong NOVA1 expression in T lymphocytes and stromal spindle cells. (**G**,**H**) Almost all tumor cells, T lymphocytes, and stromal spindle cells are negative for NOVA1. Only a few stromal spindle cells reveal occasional NOVA1 expression of moderate intensity. (**I**) Most tumor cells show negative to weak NOVA1 expression. Intratumoral and peritumoral T lymphocytes, as well as stromal spindle cells, frequently exhibit attenuated NOVA1 expression. All figures are captured at x400 magnification.

Expression of NOVA1 was scored respectively in tumor cells, stromal spindle cells (fibroblasts, support cells, and endothelial cells), and immune cells (T lymphocytes) (Fig. 6). Nuclear expression of NOVA1, Twist, and SNAI1/SLUG were analyzed according to the semi-quantitative H-score method: this method yields a total score range of 0 to 300^33^, which is obtained by multiplying the dominant nuclear staining intensity score (0, no staining; 1, weak or barely detectable nuclear staining; 2, distinct brown nuclear staining; 3, strong dark brown nuclear staining) by the percentage (0–100%) of positive cells. Densities of tumor infiltrating T lymphocytes (TILs) were semiquantitatively scored as follows^28,34^: the five most representative sections at high-power magnification (x400) were selected. Intact lymphocytes expressing CD3 and CD8 were counted manually, and the numbers of counted cells were averaged (Supplementary Fig. S10A,B). Conventionally accepted criteria for HPV-positivity were used for p16 IHC, and positivity was defined as the presence of strong and diffuse nuclear and cytoplasmic staining in >70% of the HNSCC cells. All other staining patterns were scored as negative (Supplementary Fig. S10C)^35^.

### Gene expression profile analysis

Gene expression profiles with normalized RSEM values from the TCGA-HNSC cohort (https://cancergenome.nih.gov, n = 348) were downloaded using the TCGAbiolinks R package and were analyzed. Samples were classified into upper one-third and lower one-third sample groups according to expression levels of *NOVA1*. Immune and stromal cell abundance in individual specimens was calculated by the Microenvironment Cell Populations-counter (MCP-counter) R package^36^, and the Wilcoxon Rank-Sum test was performed to determine differences in cell abundance between the two groups using R software.

### Statistical analysis

The Mann-Whitney U test, one-way ANOVA, two-sample t-test, X^2^ test, and Pearson’s or Spearman’s correlation test were conducted to analyze the significance of differences among variables, as appropriate. Overall survival (OS) was measured from the date of surgery to the date of death or last follow-up visit. Progression-free survival (PFS) was measured from the date of initial diagnosis until disease progression, defined as cancer recurrence, continuance of progressive disease without complete remission, or cancer-related death, during the study period. Patient survival rates were determined using the Kaplan-Meier method, and differences in survival rates were compared using the log-rank test. Multivariate analysis was performed using the Cox proportional hazards model. A two-sided p < 0.05 was considered statistically significant. Statistical analyses were performed using IBM SPSS 22 software for Windows (IBM Corp, Somers, New York).

## Supplementary information

Supplementary figures and tables

## Data Availability

The datasets used and/or analyzed in this study are available from the corresponding author upon reasonable request.

## References

[1] Barash Y (2010). Deciphering the splicing code. Nature.

[2] de la Grange P, Gratadou L, Delord M, Dutertre M, Auboeuf D (2010). Splicing factor and exon profiling across human tissues. Nucleic Acids Res.

[3] Merkin J, Russell C, Chen P, Burge CB (2012). Evolutionary dynamics of gene and isoform regulation in Mammalian tissues. Science.

[4] Kalsotra A, Cooper TA (2011). Functional consequences of developmentally regulated alternative splicing. Nat Rev Genet.

[5] Mallinjoud P (2014). Endothelial, epithelial, and fibroblast cells exhibit specific splicing programs independently of their tissue of origin. Genome Res.

[6] Ludlow AT (2018). NOVA1 regulates hTERT splicing and cell growth in non-small cell lung cancer. Nat Commun.

[7] Yu X, Zheng H, Chan MTV, Wu WKK (2018). NOVA1 acts as an oncogene in melanoma via regulating FOXO3a expression. J Cell Mol Med.

[8] Zhang YA (2016). RNA binding protein Nova1 promotes tumor growth *in vivo* and its potential mechanism as an oncogene may due to its interaction with GABAA Receptor-gamma2. J Biomed Sci.

[9] Kim EK (2017). Implications of NOVA1 suppression within the microenvironment of gastric cancer: association with immune cell dysregulation. Gastric cancer.

[10] Kim EK (2016). Upregulated neuro-oncological ventral antigen 1 (NOVA1) expression is specific to mature and immature T-and NK-cell lymphomas. J Pathol Transl Med.

[11] Yoon SO (2016). NOVA1 inhibition by miR-146b-5p in the remnant tissue microenvironment defines occult residual disease after gastric cancer removal. Oncotarget.

[12] Buckley CD, Barone F, Nayar S, Benezech C, Caamano J (2015). Stromal cells in chronic inflammation and tertiary lymphoid organ formation. Annu Rev Immunol.

[13] Dieu-Nosjean MC, Goc J, Giraldo NA, Sautes-Fridman C, Fridman WH (2014). Tertiary lymphoid structures in cancer and beyond. Trends Immunol.

[14] Di Caro G (2015). Tertiary lymphoid tissue in the tumor microenvironment: from its occurrence to immunotherapeutic implications. Int Rev Immunol.

[15] Drayton DL, Liao S, Mounzer RH, Ruddle NH (2006). Lymphoid organ development: from ontogeny to neogenesis. Nat Immunol.

[16] Ruddle NH, Akirav EM (2009). Secondary lymphoid organs: responding to genetic and environmental cues in ontogeny and the immune response. J Immunol.

[17] Ndiaye C (2014). HPV DNA, E6/E7 mRNA, and p16INK4a detection in head and neck cancers: a systematic review and meta-analysis. Lancet Oncol.

[18] Shi CS (2012). Activation of autophagy by inflammatory signals limits IL-1beta production by targeting ubiquitinated inflammasomes for destruction. Nat Immunol.

[19] Ishii KJ (2006). A Toll-like receptor-independent antiviral response induced by double-stranded B-form DNA. Nat Immunol.

[20] Yang P (2010). The cytosolic nucleic acid sensor LRRFIP1 mediates the production of type I interferon via a beta-catenin-dependent pathway. Nat Immunol.

[21] Jones JW (2010). Absent in melanoma 2 is required for innate immune recognition of Francisella tularensis. Proc Natl Acad Sci USA.

[22] Kohlhapp FJ, Mitra AK, Lengyel E, Peter ME (2015). MicroRNAs as mediators and communicators between cancer cells and the tumor microenvironment. Oncogene.

[23] Ferris RL (2015). Immunology and Immunotherapy of Head and Neck Cancer. J Clin Oncol.

[24] Coffelt SB, de Visser KE (2015). Immune-mediated mechanisms influencing the efficacy of anticancer therapies. Trends Immunol.

[25] Topalian SL, Drake CG, Pardoll DM (2015). Immune checkpoint blockade: a common denominator approach to cancer therapy. Cancer Cell.

[26] Cho YA (2016). Alteration status and prognostic value of MET in head and neck squamous cell carcinoma. J Cancer.

[27] Cho Yoon Ah, Chung Ji Myung, Ryu Hyunmi, Kim Eun Kyung, Cho Byoung Chul, Yoon Sun Och (2019). Investigating Trk Protein Expression between Oropharyngeal and Non-oropharyngeal Squamous Cell Carcinoma: Clinical Implications and Possible Roles of Human Papillomavirus Infection. Cancer Research and Treatment.

[28] Ryu HJ (2017). Architectural patterns of p16 immunohistochemical expression associated with cancer immunity and prognosis of head and neck squamous cell carcinoma. APMIS.

[29] Kim HR (2016). PD-L1 expression on immune cells, but not on tumor cells, is a favorable prognostic factor for head and neck cancer patients. Sci Rep.

[30] Ryu HJ, Kim EK, Cho BC, Yoon SO (2018). Characterization of head and neck squamous cell carcinoma arising in young patients: Particular focus on molecular alteration and tumor immunity. Head Neck.

[31] Edge, S. B. *et al*. Editor. *AJCC cancer staging manual*. 7^th^ ed (Springer, New York, 2010).

[32] Barnes, L., Eveson, J. W., Reichart, P., Sidransky, D. Editor. *World Health Organization Classification of Head and Neck Tumours. Pathology and Genetics of Head and Neck Tumours*. 3^rd^ ed. (IARC Press, Lyon, 2005).

[33] Park E (2015). Membranous Insulin-like Growth Factor-1 Receptor (IGF1R) Expression Is Predictive of Poor Prognosis in Patients with Epidermal Growth Factor Receptor (EGFR)-Mutant Lung Adenocarcinoma. J Pathol Transl Med.

[34] Balermpas P (2016). CD8+ tumour-infiltrating lymphocytes in relation to HPV status and clinical outcome in patients with head and neck cancer after postoperative chemoradiotherapy: A multicentre study of the German cancer consortium radiation oncology group (DKTK-ROG). Int J Cancer.

[35] Ang KK (2010). Human papillomavirus and survival of patients with oropharyngeal cancer. N Engl J Med.

[36] Becht E (2016). Estimating the population abundance of tissue-infiltrating immune and stromal cell populations using gene expression. Genome Biol.

