# Author Correction: NOVA1 induction by inflammation and NOVA1 suppression by epigenetic regulation in head and neck squamous cell carcinoma

**DOI:** 10.1038/s41598-020-77481-5

**Published:** 2020-11-30

**Authors:** Eun Kyung Kim, Yoon Ah Cho, Mi-kyoung Seo, Hyunmi Ryu, Byoung Chul Cho, Yoon Woo Koh, Sun Och Yoon

**Affiliations:** 1 Department of Pathology, Severance Hospital, Yonsei University College of Medicine, Seoul, 03722 South Korea; 2 Department of Pathology, National Health Insurance Service Ilsan Hospital, Goyang, 10444 South Korea; 3 Department of Biomedical Systems Informatics, Brain Korea 21 PLUS Project for Medical Science, Yonsei University College of Medicine, Seoul, 03722 South Korea; 4 Division of Medical Oncology, Department of Internal Medicine, Yonsei Cancer Center, Severance Hospital, Yonsei University College of Medicine, Seoul, 03722 South Korea; 5 Department of Otorhinolaryngology, Severance Hospital, Yonsei University College of Medicine, Seoul, 03722 South Korea

Correction to: *Scientific Reports* 10.1038/s41598-019-47755-8, published online 2 August 2019

This Article contains errors in the Method section under subheading ‘Clinical samples’.

“A written informed consent of the patients was obtained, samples and clinical information were anonymized.”

should read:

“A written informed consent of the patients was waived, samples and clinical information were anonymized.”

