# Supplementary material for: NOVA1 induction by inflammation and NOVA1 suppression by epigenetic regulation in head and neck squamous cell carcinoma
Source: Sci Rep. 2019 Aug 2;9:11231. doi: 10.1038/s41598-019-47755-8 (PMC6677744; doi:10.1038/s41598-019-47755-8)
Supplement: Supplementary file 1 — Supplementary figures and tables [file 41598_2019_47755_MOESM1_ESM.pdf]

Supplementary Information

NOVA1 induction by inflammation and NOVA1 suppression by epigenetic regulation in head and neck squamous cell carcinoma

Eun Kyung Kim[1,2], Yoon Ah Cho[1], Mi-kyoung Seo[3], Hyunmi Ryu[1], Byoung Chul Cho[4], Yoon Woo Koh[5], Sun Och Yoon[1*]

[1]Department of Pathology, Severance Hospital, Yonsei University College of Medicine, Seoul, 03722, Republic of Korea. [2]Department of Pathology, National Health Insurance Service Ilsan Hospital, Goyang, 10444, Republic of Korea. [3]Department of Biomedical Systems Informatics, Brain Korea 21 PLUS Project for Medical Science, Yonsei University College of Medicine, Seoul, 03722, Republic of Korea. [4]Division of Medical Oncology, Department of Internal Medicine, Yonsei Cancer Center, Severance Hospital, Yonsei University College of Medicine, Seoul, 03722, Republic of Korea. [5]Department of Otorhinolaryngology, Severance Hospital, Yonsei University College of Medicine, Seoul, 03722, Republic of Korea

Corresponding Author

Sun Och Yoon, Department of Pathology, Severance Hospital, Yonsei University College of Medicine, 50-1 Yonsei-ro, Seodaemun-gu, Seoul, 03722, Republic of Korea. Phone: 82-2-2228-1763; Fax: 82-2-362-0860; E-mail: soyoon@yuhs.ac

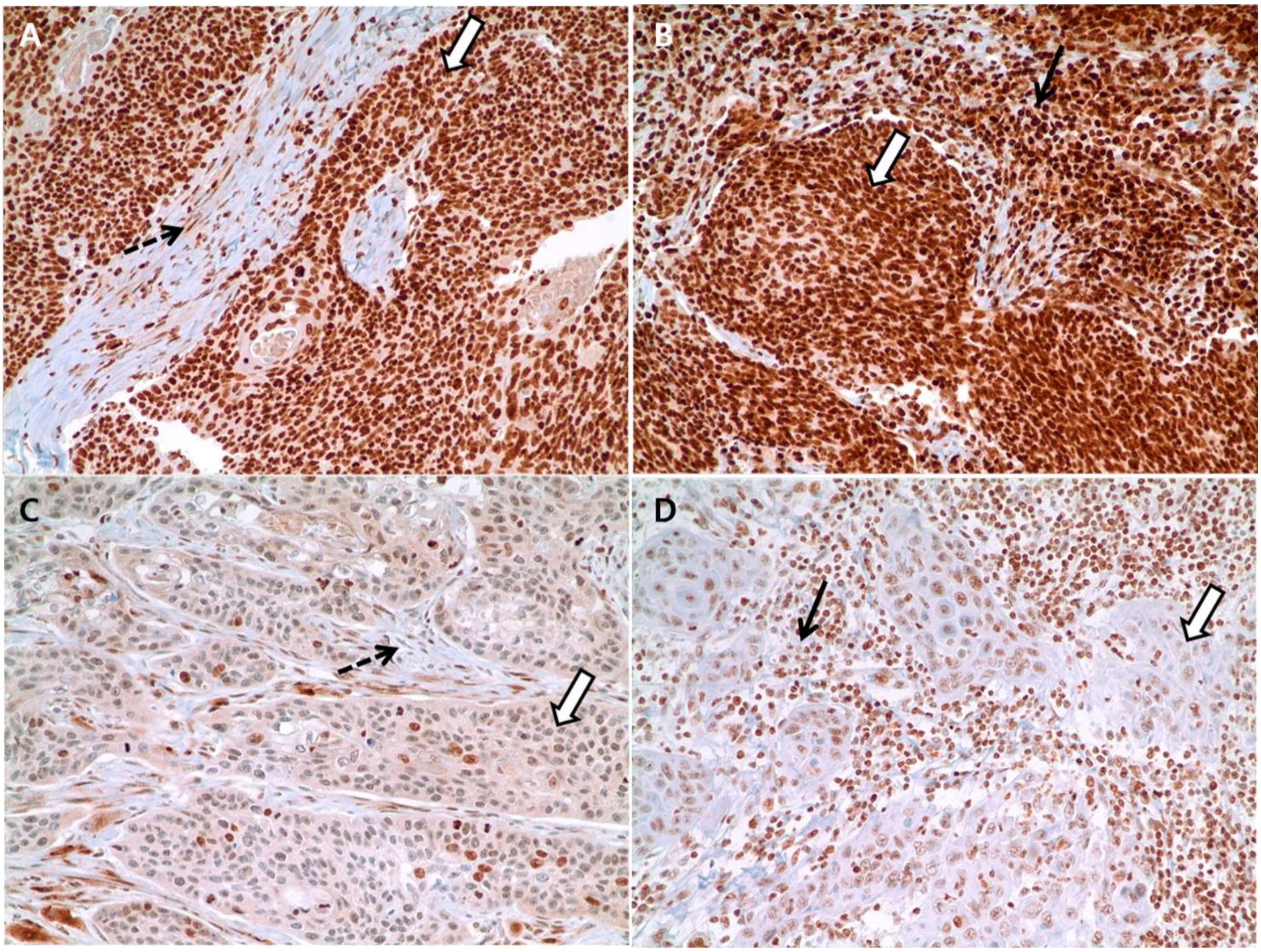

Supplementary Figure S1. NOVA1 expression in head and neck squamous cell carcinoma. (A and B) Strong and diffuse NOVA1 expression in the nuclei of tumor cells (empty arrow), T lymphocytes (black arrow), and stromal spindle cells/fibroblasts (dotted arrow) is noted within the tissue microenvironment of HPV-mediated oropharyngeal squamous cell carcinoma. (C and D) Attenuated NOVA1 expression is noted in cells within the tissue microenvironment of HPV-negative, non-oropharyngeal squamous cell carcinoma.

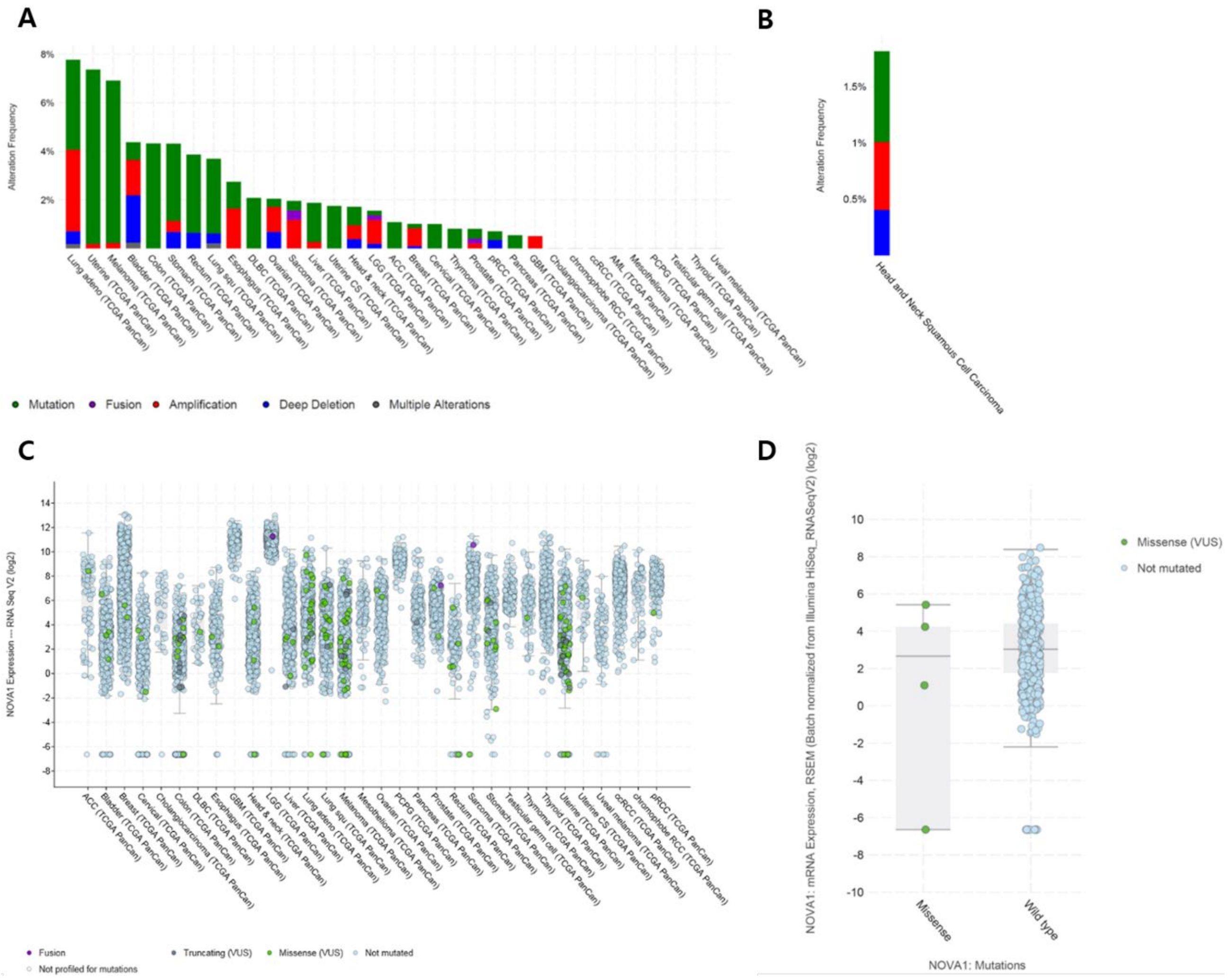

Supplementary Figure S2. Genetic alteration of NOVA1 in TCGA (PanCancer Atlas; http://www.cbioportal.org)[1,2]. (A and B) Genetic alteration of NOVA1 is noted in 2.4% of all tumors (n=10967) and only 1.72% of head and neck squamous cell carcinomas (n=523). (C and D) NOVA1 expression status varies within the same cohorts.

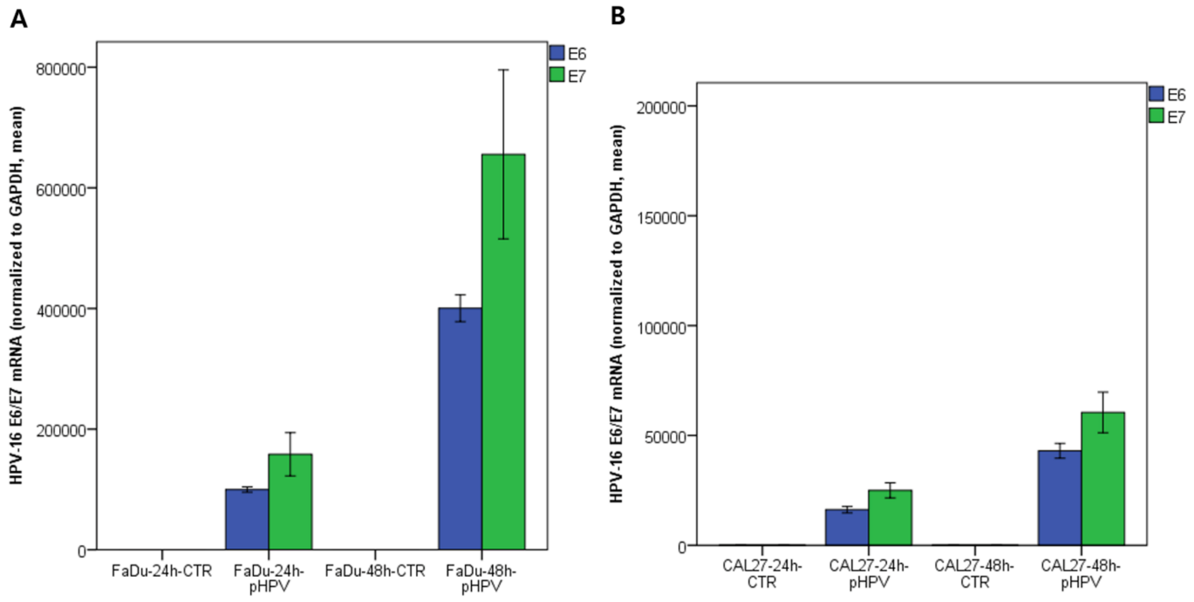

Supplementary Figure S3. Transfection of p1321 HPV-16 E6/E7 plasmid. (A and B) After transfection into FaDu and CAL27 cells, E6 and E7 gene expression is noted.

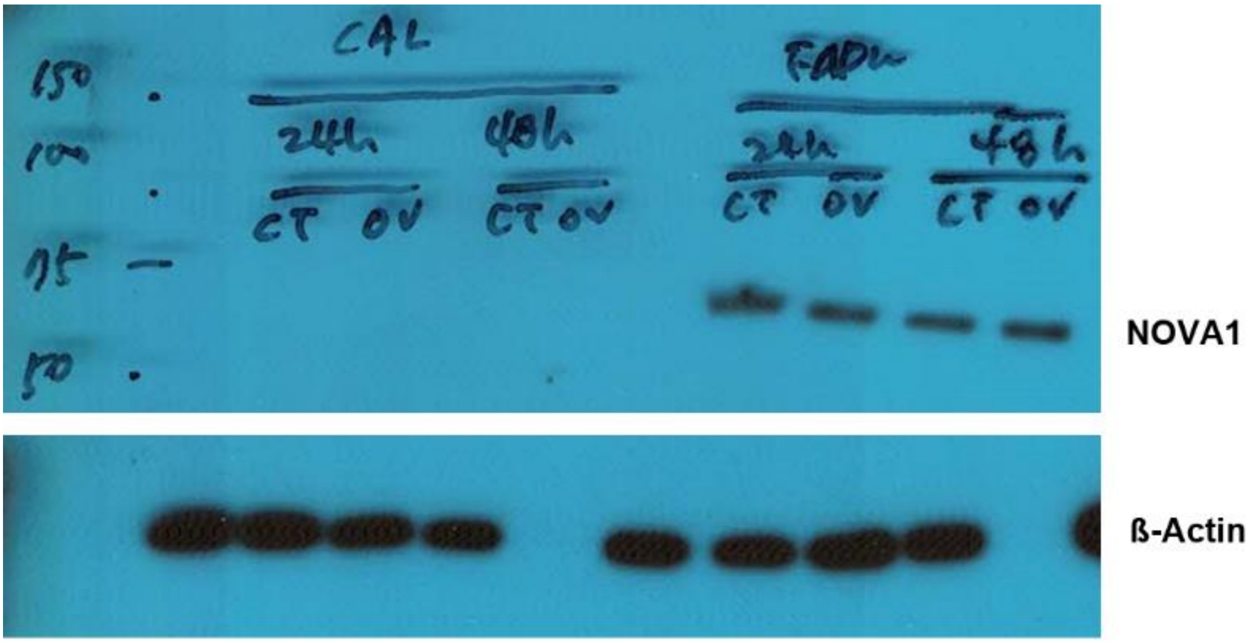

Supplementary Figure S4. Original pictures from Western blot membranes. NOVA1 protein expression was detected in FaDu cells, but not in CAL27 cells. Transfection of HPV type 16 *E6/E7* genes into FaDu and CAL27 did not induce a significant change in NOVA1 protein expression. These cropped blots are used in the main figure (Figure 1A).

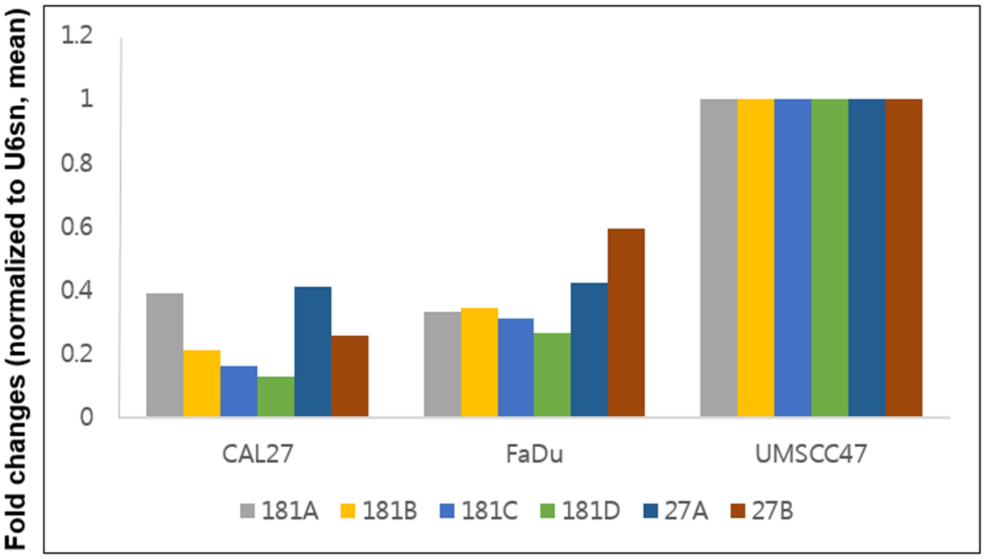

Supplementary Figure S5. Endogenous expression of predicted target miRs (miR-181a, miR-181b, miR-181c, miR-181d, miR-27a, and miR-27b) in the HPV-negative non-oropharynx SCC cell lines FaDu and CAL27 and the HPV-positive oropharynx SCC cell line UMSCC47. Fold changes in each molecule were calculated in comparison to UMSCC47.

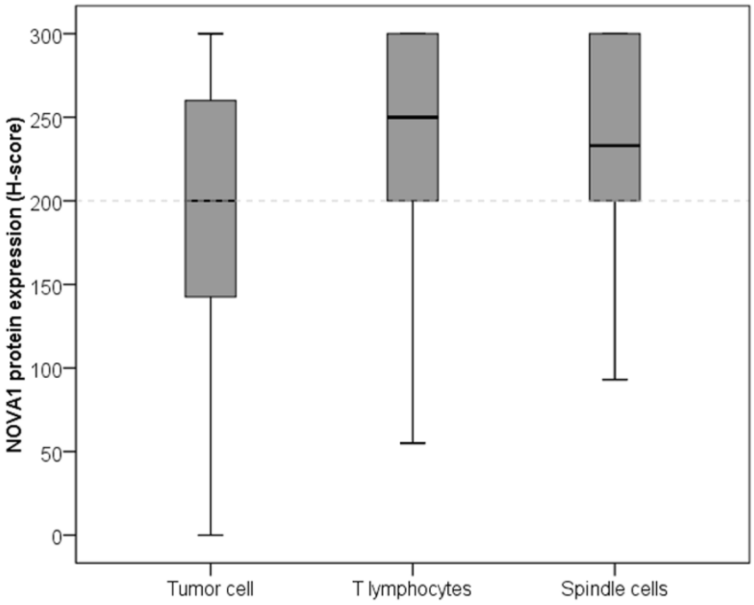

Supplementary Figure S6. NOVA1 expression levels in tumor cells and microenvironment cells in HNSCC. Lymphocytes and stromal spindle cells generally showed diffuse and strong expression of NOVA1, while attenuated NOVA1 expression (H-score <200, representing a loss of NOVA1 in more than one-third of cells) was frequently observed in tumor cells.

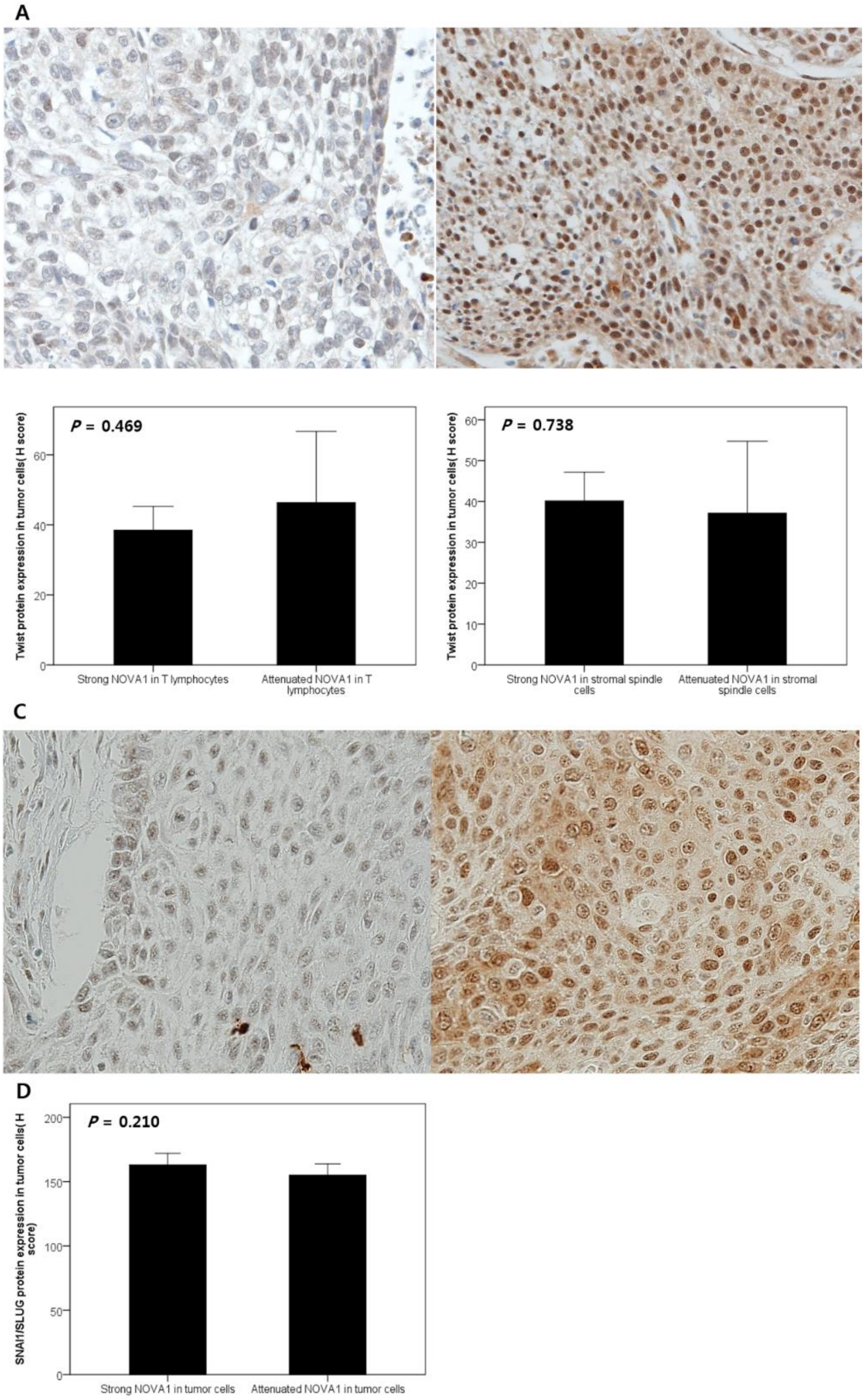

Supplementary Figure S7. NOVA1 expression in association with EMT markers. (A) Representative cases of low and high Twist expression in tumor cells. (B) Twist expression did not show a significant difference according to NOVA1 status in T lymphocytes or stromal spindle cells/fibroblasts. (C) Representative cases of low and high SNAI1/SLUG expression in tumor cells. (D) SNAI1/SLUG expression in tumor cells did not show a significant difference according to tumor NOVA1 expression status.

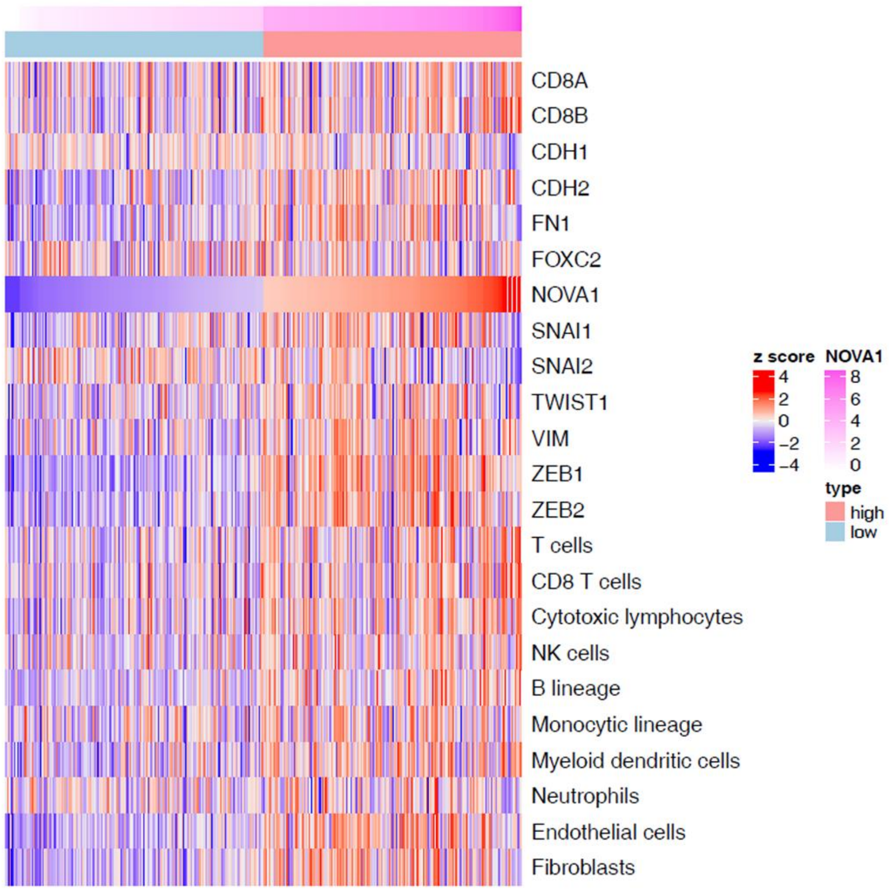

Supplementary Figure S8. Microenvironment Cell Populations-counter analysis. The z-score transformed values of log2 (normalized rsem+1) values of genes and MCP-counter values were used to identify differences in cell type abundance, inflammation-related genes, and EMT-related genes, respectively, between groups. Lower abundance of immune cell and stromal cell populations, downregulation of CD8+T cell-related genes, downregulation of TWIST and SNAI1, and upregulation of SNAI2 and TGFB1 were related to low NOVA1 expression.

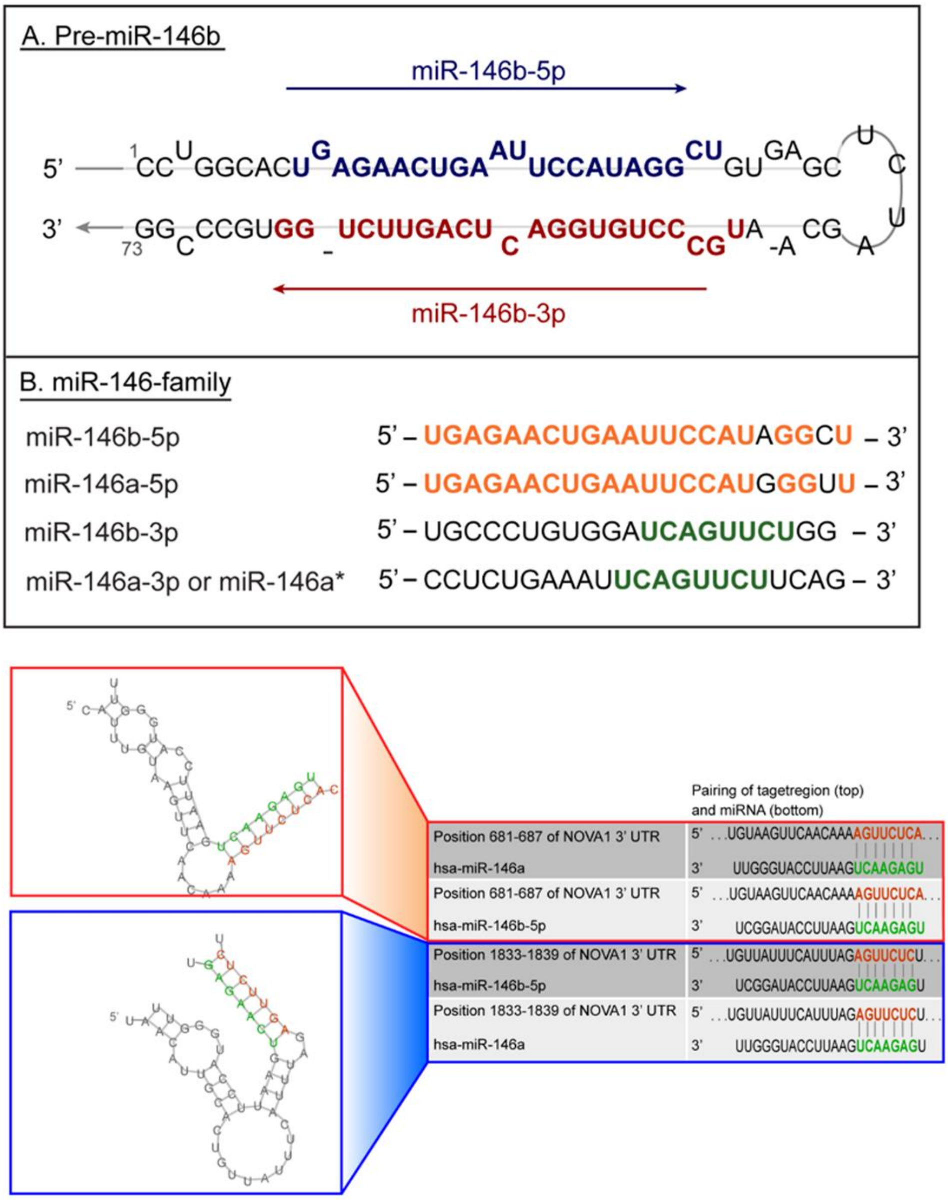

Supplementary Figure S9. Sequences of miR-146. The sequences of miR-146a-5p and miR-146b-5p are very similar and their predicted target sites of NOVA1 are identical (-AGUUCUC-).

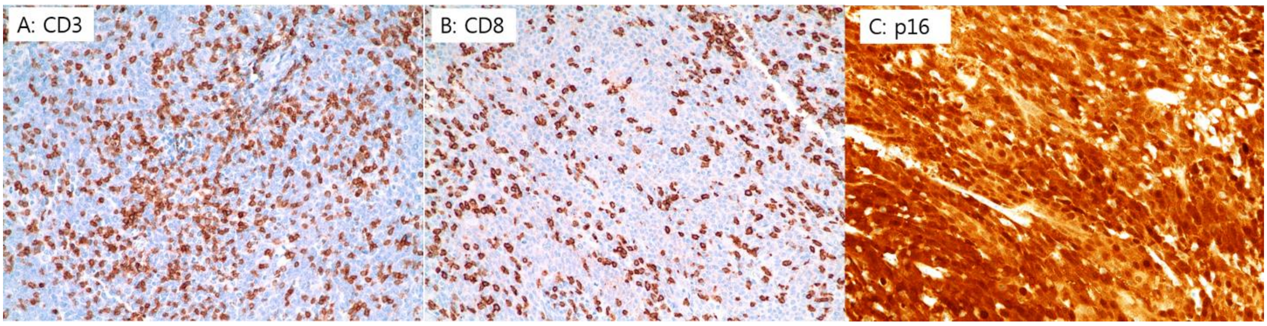

Supplementary Figure S10. Representative cases showing high densities of intraepithelial T lymphocytes and p16 positivity. A and B, Most T lymphocytes are positive for CD3 and CD8. C. A representative case showing positive p16 expression in tumor cells.

Supplementary Table S1. Clinicopathologic characteristics for radically resected tissue samples of head and neck squamous cell carcinoma

	N	(%)
All	396	
Sex		
Male	298	75.3
Female	98	24.7
Age		
Median (range), yr	58 (22-88)	
Smoking		
Never smoker	154	38.9
Ex-smoker	80	20.2
Current smoker	162	40.9
Alcohol		
No	141	35.6
Yes	255	64.4
Anatomical sites		
Oropharynx	116	29.3
Non-oropharynx	280	70.7
p16 immunohistochemistry		
Negative	271	68.4
Positive	122	30.8
Not evaluable[a]	3	0.8
Lymphovascular invasion		
Negative	320	80.8
Positive	76	19.2
Perineural invasion		
Negative	342	86.4
Positive	54	13.6
Resection margin		
Negative	305	77
Positive	91	23
pT category[b]		
pT1-2	326	82.3
pT3-4	70	17.7

pN category[b]		
pN0-1	248	62.6
pN2-3	148	37.4
Postoperative adjuvant therapy[c]		
No treatment	145	36.6
RTx alone	150	37.9
CTx alone or other	2	0.5
CCRT	99	25
Progression		
No	293	74
Yes	103	26
Death		
No	324	81.8
Yes	72	18.2

[a]Information for p16 expression status was unavailable due to inadequate immunohistochemical quality

[b]Pathologic T and N stages were categorized according to 7th AJCC guidelines.

[c]Incomplete CCRT was done for a case. RTx means radiation therapy; CTx, chemotheray; CCRT, chemoradiation therapy

Supplementary Table S2. Sequence of each primer

Gene name	Forward	Reverse
HPV type 16 *E6*	CTGCAATGTTTCAGGACCCA	TCATGTATAGTTGTTTGCAGCTCTGT
HPV type 16 *E7*	AAGTGTGACTCTACGCTTCGGTT	GCCCATTAACAGGTCTTCCAAA
NOVA1	TTGCCATCTTCCCCAACTAC	TTACAGCCTTCACAGTAGCAC
TWIST1	CGGGAGTCCGCAGTCTTA	TGAATCTTGCTCAGCTTGTC
SNAI1	GAGGCGGTGGCAGACTAG	GACACATCGGTCAGACCAG

Supplementary Table S3. NOVA1 protein expression status for 50 head and neck squamous cell carcinomas

Case No.	Site	NOVA1 expression (H-score)		
		Tumor cells	T lymphocytes	Stromal spindle cells
1	non-oropharynx	10	45	45
2	non-oropharynx	15	35	120
3	non-oropharynx	193	233	227
4	non-oropharynx	165	160	180
5	non-oropharynx	110	220	230
6	non-oropharynx	47	267	200
7	non-oropharynx	15	180	135
8	oropharynx	3	3	3
9	non-oropharynx	40	15	25
10	non-oropharynx	125	250	250
11	non-oropharynx	83	300	300
12	oropharynx	167	233	200
13	oropharynx	127	233	233
14	non-oropharynx	100	300	250
15	non-oropharynx	67	120	120
16	oropharynx	30	80	15
17	oropharynx	100	227	193
18	oropharynx	33	80	120
19	non-oropharynx	145	180	200
20	non-oropharynx	127	200	200
21	non-oropharynx	70	100	30
22	oropharynx	200	220	160
23	non-oropharynx	117	220	207
24	non-oropharynx	0	0	0
25	non-oropharynx	55	200	180
26	non-oropharynx	60	200	200
27	oropharynx	85	200	200
28	non-oropharynx	0	5	5
29	non-oropharynx	150	300	200
30	non-oropharynx	80	150	150
31	non-oropharynx	120	200	200
32	non-oropharynx	15	10	200
33	non-oropharynx	0	27	37
34	non-oropharynx	97	233	193
35	oropharynx	15	20	105
36	non-oropharynx	80	230	240
37	non-oropharynx	135	150	150
38	non-oropharynx	33	67	93
39	non-oropharynx	70	233	233

40	oropharynx	40	85	45
41	non-oropharynx	107	167	153
42	non-oropharynx	100	200	200
43	non-oropharynx	100	250	200
44	non-oropharynx	65	125	125
45	non-oropharynx	35	200	200
46	oropharynx	80	153	160
47	non-oropharynx	200	300	300
48	oropharynx	0	0	0
49	non-oropharynx	240	260	267
50	non-oropharynx	180	300	250

Supplementary Table S4. Sequences of primers for each miR

	mature microRNA	RT primer	PCR primer	
			miR-specific forward primer	universal reverse primer
hsa-miR-146a-5p	UGAGAACUGAAUUCCAUGGGUU	GTCGTATCCAGTGCAGGGTCCGAGGTATTCGCACTGGATACGACAACCCA	AAAAAAAAAAGCCCGCTGAGAACTGAATTCCA	AAAAAAAAAAGTGCAGGGTCCGAGGT
hsa-miR-146b-5p	UGAGAACUGAAUUCCAUAGGCU	GTCGTATCCAGTGCAGGGTCCGAGGTATTCGCACTGGATACGACAGCCTA	AAAAAAAAAAGCCCGCTGAGAACTGAATTCCA	AAAAAAAAAAGTGCAGGGTCCGAGGT
hsa-miR-27a-3p	UUCACAGUGGCUAAGUUCCGC	GTCGTATCCAGTGCAGGGTCCGAGGTATTCGCACTGGATACGACGCGGAA	AAAAAAAAAAGCCCGCTTCACAGTGGCTAAG	AAAAAAAAAAGTGCAGGGTCCGAGGT
hsa-miR-27b-3p	UUCACAGUGGCUAAGUUCUGC	GTCGTATCCAGTGCAGGGTCCGAGGTATTCGCACTGGATACGACGCAGAA	AAAAAAAAAAGCCCGCTTCACAGTGGCTAAG	AAAAAAAAAAGTGCAGGGTCCGAGGT
hsa-miR-181a-5p	AACAUUCAACGCUGUCGGUGAGU	GTCGTATCCAGTGCAGGGTCCGAGGTATTCGCACTGGATACGACACTCAC	AAAAAAAAAAGCCCGCAACATTCAACGCTGTCG	AAAAAAAAAAGTGCAGGGTCCGAGGT
hsa-miR-181b-5p	AACAUUCAUUGCUGUCGGUGGGU	GTCGTATCCAGTGCAGGGTCCGAGGTATTCGCACTGGATACGACACCCAC	AAAAAAAAAAGCCCGCAACATTCATTGCTGTCG	AAAAAAAAAAGTGCAGGGTCCGAGGT
hsa-miR-181c-5p	AACAUUCAACCUGUCGGUGAGU	GTCGTATCCAGTGCAGGGTCCGAGGTATTCGCACTGGATACGACACTCAC	AAAAAAAAAAGCCCGCAACATTCAACCTGTCG	AAAAAAAAAAGTGCAGGGTCCGAGGT
hsa-miR-181d-5p	AACAUUCAUUGUUGUCGGUGGGU	GTCGTATCCAGTGCAGGGTCCGAGGTATTCGCACTGGATACGACACCCAC	AAAAAAAAAAGCCCGCAACATTCATTGTTGTCG	AAAAAAAAAAGTGCAGGGTCCGAGGT
U6sn	CUGCGCAAGGAUGACACGCAAAUUCGUGAAGCGUUCCAUAUUUUU	GTCGTATCCAGTGCAGGGTCCGAGGTATTCGCACTGGATACGACTCACGA	AAAAAAAAAAGCCCGCCTGCGCAAGGATGAC	AAAAAAAAAAGTGCAGGGTCCGAGGT

Supplementary Table S5. Univariate and multivariate Cox proportional hazards regression analyses for overall survival

		Univariate				Multivariate			
		P value	HR	95% CI		*P* value	HR	95% CI	
Sex	Male vs Female	0.917	1.029	0.603	1.755	NA			
Age (median: 58)	**≥ 58 vs < 58**	**0.015**	**1.813**	**1.121**	**2.934**	**0.018**	**1.842**	**1.111**	**3.053**
Smoking	Ex-smocker vs Never smocker	0.842	0.933	0.47	1.85	NA			
	Current vs Never	0.302	1.309	0.785	2.182	NA			
Anatomical sites	**Non-oropharynx vs Oropharynx**	**0.003**	**2.783**	**1.427**	**5.427**	**0.038**	**2.594**	**1.055**	**6.38**
p16	**Negative vs Positive**	**0.004**	**2.46**	**1.323**	**4.573**	0.544	1.277	0.58	2.811
LVI	**Positive vs Negative**	**0.011**	**1.936**	**1.164**	**3.221**	0.188	1.482	0.825	2.661
Perineural invasion	**Positive vs Negative**	**0.009**	**2.095**	**1.201**	**3.655**	0.485	1.24	0.678	2.267
Resection margin	**Positive vs Negative**	**0.011**	**1.884**	**1.154**	**3.077**	0.053	1.672	0.992	2.817
pT stage	**pT3-4 vs pT1-2**	**<0.001**	**3.863**	**2.41**	**6.191**	**0.001**	**2.437**	**1.444**	**4.113**
pN stage	**pN2-3 vs pN0-1**	**<0.001**	**2.688**	**1.682**	**4.296**	**<0.001**	**2.583**	**1.532**	**4.354**
NOVA1 in tumor cells	Attenuated vs Strong	**0.02**	**1.767**	**1.096**	**2.849**	**0.005**	**2.104**	**1.255**	**3.525**
NOVA1 in immune cells	Attenuated vs Strong	0.15	1.535	0.856	2.752	NA			
NOVA1 in spindle cells	Attenuated vs Strong	0.208	1.441	0.816	2.546	NA			
CD3+TILs	**Low (below mean) vs High**	**0.016**	**1.905**	**1.128**	**3.217**	0.734	0.896	0.476	1.687
CD8+TILs	**Low (below mean) vs High**	**0.001**	**2.987**	**1.607**	**5.553**	**0.034**	**2.235**	**1.064**	**4.698**

CI, confidence interval; HR, hazard ratio; LVI, lymphovascualr invasion; NA, not applicable

Supplementary Table S6. Univariate and multivariate Cox proportional hazards regression analyses for progression free survival

		Univariate				Multivariate			
		P value	HR	95% CI		*P* value	HR	95% CI	
Sex	Male vs Female	0.498	1.174	0.738	1.868	NA			
Age (median: 58)	**≥ 58 vs < 58**	**0.011**	**1.674**	**1.124**	**2.495**	**0.01**	**1.732**	**1.141**	**2.628**
Smoking	Ex-smocker vs Never smocker	0.959	1.016	0.565	1.825	0.512	0.818	0.448	1.492
	Current vs Never	**0.031**	**1.615**	**1.045**	**2.497**	0.182	1.364	0.865	2.151
Anatomical sites	**Non-oropharynx vs Oropharynx**	**<0.001**	**2.727**	**1.577**	**4.716**	**0.03**	**2.243**	**1.083**	**4.643**
p16	**Negative vs Positive**	**<0.001**	**2.558**	**1.52**	**4.305**	0.23	1.49	0.777	2.859
LVI	**Positive vs Negative**	**0.012**	**1.741**	**1.127**	**2.689**	0.175	1.409	0.858	2.314
Perineural invasion	**Positive vs Negative**	**0.002**	**2.106**	**1.324**	**3.349**	0.292	1.316	0.789	2.195
Resection margin	**Positive vs Negative**	0.092	1.447	0.942	2.224	NA			
pT stage	**pT3-4 vs pT1-2**	**<0.001**	**2.924**	**1.944**	**4.4**	**0.005**	1.881	1.21	2.924
pN stage	**pN2-3 vs pN0-1**	**<0.001**	**2.183**	**1.482**	**3.216**	**<0.001**	2.189	1.414	3.389
NOVA1 in tumor cells	Attenuated vs Strong	**0.032**	**1.537**	**1.038**	**2.274**	**0.03**	**1.599**	**1.047**	**2.44**
NOVA1 in immune cells	Attenuated vs Strong	0.918	1.029	0.595	1.78	NA			
NOVA1 in spindle cells	Attenuated vs Strong	0.291	1.301	0.798	2.12	NA			
CD3+TILs	**Low (below mean) vs High**	**0.009**	**1.77**	**1.152**	**2.72**	0.359	0.78	0.459	1.326
CD8+TILs	**Low (below mean) vs High**	**<0.001**	**2.877**	**1.73**	**4.786**	**0.008**	**2.307**	**1.247**	**4.268**

CI, confidence interval; HR, hazard ratio; LVI, lymphovascualr invasion; NA, not applicable

Supplementary Table S7. Differences in cell abundance between high NOVA1 and low NOVA1 gene expression groups in Microenvironment Cell Populations-counter (MCP-counter) analysis

Cell type	*P* value
Endothelial cells	9.26E-23
Fibroblasts	5.31E-18
B lineage	5.66E-14
Myeloid dendritic cells	3.26E-10
Cytotoxic lymphocytes	2.46E-08
T cells	2.73E-07
CD8 T cells	6.06E-06
NK cells	0.00029996
Monocytic lineage	0.001216916
Neutrophils	0.849968198

MCP-counter analysis was performed using the gene set of HNSCC cohorts generated from TCGA (https://cancergenome.nih.gov, n= 348), and the differences between groups was determined by the Wilcoxon Rank-Sum test using R software.

Supplementary Table S8. Differences in gene expression between high NOVA1 and low NOVA1 gene expression groups in Microenvironment Cell Populations-counter (MCP-counter) analysis

Gene	*P* value
NOVA1	1.52E-58
ZEB1	8.63E-31
ZEB2	1.07E-20
CDH2	5.18E-13
IL1A	1.98E-12
FN1	4.57E-11
VIM	9.76E-10
SNAI1	2.82E-07
IL1B	2.20E-06
IL10	5.72E-06
CD8B	6.06E-06
TWIST	6.53E-06
TNFRSF1A	5.00E-05
TGFB1	9.46E-05
SNAI2	0.002075
CD8A	0.007571
VEGFA	0.010982
CDH1	0.012472
IL4	0.104415
IL6	0.707174
TNF	0.733892
FOXC2	0.821674

MCP-counter analysis was performed using the gene set of HNSCC cohorts generated from TCGA (https://cancergenome.nih.gov, n= 348), and the differences between groups was determined by the Wilcoxon Rank-Sum test using R software.

Reference

1. Gao, J. *et al.* Integrative analysis of complex cancer genomics and clinical profiles using the cBioPortal. *Sci Signal* **6**, pl1 (2013).
2. Cerami, E. *et al.* The cBio cancer genomics portal: an open platform for exploring multidimensional cancer genomics data. *Cancer Discov* **2**, 401-404 (2012).
